# Carotid Atherosclerosis as a Surrogate Maker of Cardiovascular Disease in Diabetic Patients

**DOI:** 10.1155/2013/979481

**Published:** 2013-11-10

**Authors:** Ryuichi Kawamoto, Tateaki Katoh, Tomo Kusunoki, Nobuyuki Ohtsuka

**Affiliations:** ^1^Department of Community Medicine, Ehime University Graduate School of Medicine, Ehime, Japan; ^2^Department of Internal Medicine, Seiyo Municipal Nomura Hospital, 9-53 Nomura, Nomura-cho, Seiyo, Ehime 797-1212, Japan

## Abstract

Many studies have shown that carotid intima-media thickness (IMT) is associated with cardiovascular disease (CVD). Although it remains inconclusive whether assessment of carotid IMT is useful as a screening test for CVD in Japanese diabetic patients, a total of 271 patients (151 men aged 66 ± 10 (standard deviation) years and 220 women aged 71 ± 8 years) were divided into two groups based on the presence of CVD. We cross-sectionally assessed the ability of carotid IMT to identify CVD corresponding to treatment that was examined by receiver-operating characteristic (ROC) curve analyses. Among the 271 diabetic patients, 199 non-CVD and 72 CVD patients were examined. Multiple linear regression analysis using the presence of CVD as an objective variable showed that carotid IMT (*β* = 0.259, *P* < 0.001) as well as other confounding factors was a significant independent contributing factor. The ROC curve analysis showed that the best marker of CVD was carotid IMT, with an area under the ROC curve of 0.718 (95% confidence interval (CI), 0.650–0.785). The greatest sensitivity and specificity were obtained when the cut-off value of mean carotid IMT was set at 0.95 mm (sensitivity = 0.71, specificity = 0.60, and accuracy = 0.627). Our study suggests that carotid IMT may be useful for screening diabetic patients with CVD.

## 1. Introduction

Diabetes is associated with a high risk of cardiovascular disease (CVD) which is the most common cause of mortality in people with diabetes. CVD accounts for more than 70% of deaths in people with diabetes [1]. A two- to fourfold increased risk of CVD in people with diabetes compared without diabetes has been reported by various research groups [2–4].

Carotid intima-media thickness (IMT) can now be measured noninvasively by B-mode ultrasonography and is an important and sensitive surrogate marker of cardiovascular disease (CVD). A lot of evidence has shown close associations between this parameter and conventional cardiovascular risk factors, including age, obesity, smoking status, hypertension, and dyslipidemia including hypertriglycerides, low high-density lipoprotein cholesterol (HDL-C) level, increased low-density lipoprotein cholesterol (LDL-C) level, and diabetes [5, 6]. In prospective studies, carotid IMT predicted clinical CVD events independently of traditional risk factors [7–10]. Recent findings have suggested that IMT measurements can be used to stratify patients into high-risk groups [11], but meta-analyses have found that carotid IMT is not predictive of cardiovascular events [12, 13], and in 2010 the American Heart Association and the American College of Cardiology advocated the assessment of carotid IMT on intermediate risk patients if usual risk classification was not useful [14]. Thus, to our knowledge there have been few published reports assessing carotid IMT as a screening test for CVD among Japanese diabetic patients. 

We cross-sectionally examined the association of the carotid IMT and various risk factors with CVD in diabetic patients.

## 2. Methods

### 2.1. Subjects

Patients for this investigation were recruited from among consecutive diabetic patients aged ≤80 years that visited the medical department of Seiyo Municipal Nomura Hospital. Patients with severe cardiorenal (e.g., symptomatic chronic heart failure or chronic kidney disease) or nutritional disorders (albumin <2.5 g/dL) that would affect blood pressure, lipid, and glucose metabolism were excluded. Thus, 271 diabetic patients were enrolled in the study. All procedures were approved by the Ethics Committee of Seiyo Municipal Nomura Hospital, and written informed consent was obtained from each patient.

### 2.2. Case Definitions

Prevalent CVD was defined as doctor-diagnosed transient ischemic attack, cerebral infarction, ischemic heart disease, or myocardial infarction. Clinical syndrome of ischemic stroke or ischemic heart disease was defined as rapidly developing clinical symptoms and/or signs of focal and at times global loss of brain or heart function, with symptoms of or leading to earlier death, and with no apparent cause other than that of vascular origin. Patients were divided into two groups based on the presence of CVD.

### 2.3. Evaluation of Risk Factors

Information on demographic characteristics and risk factors was collected using the clinical files in all cases. Body mass index (BMI) was calculated by dividing weight (in kilograms) by the square of the height (in meters). We measured blood pressure (BP) in the right upper arm of patients in a sedentary posture using a standard sphygmomanometer or an automatic oscillometric BP recorder. Smoking status was defined as the number of cigarette packs per day multiplied by the number of years smoked (pack·year). Histories of antihypertensive and lipid-lowering medication used were also evaluated. Total cholesterol (T-C), triglycerides (TG), HDL-C, fasting plasma glucose (FPG), and creatinine (Cr) were measured under a fasting condition. Estimated Glomerular Filtration Rate (eGFR) was calculated using the following equation: 194 × Cr^−1.094^ × Age^−0.287^ × 0.739 (if female) [15]. 

#### 2.3.1. Ultrasound Image Analysis

An ultrasonograph (Hitachi EUB-565, Aloka SSD-2000, or Prosound-*α*6) equipped with a 7.5 MHz linear type B-mode probe was used by a specialist in ultrasonography to evaluate sclerotic lesions of the common carotid arteries on a day close to the day of blood biochemistry analysis (within 2 days). The ultrasonograph measurements were performed by a physician (R. K.) and the method provided a high reproducibility, with interobserver variability of 9.2%. Patients were asked to assume a supine position, and the bilateral carotid arteries were observed obliquely from the anterior and posterior directions. We measured the thickness of the intima-media complex (IMT) on the far wall of the bilateral common carotid artery about 10 mm proximal to the bifurcation of the carotid artery (as the image at that site is more clearly depicted than that at the near wall) [16, 17] as well as the wall thickness near the 10 mm point on a B-mode monitor. We then used the mean value for analysis.

### 2.4. Metabolic Syndrome (MetS)

We applied condition-specific cutoff points for MetS based on the modified criteria of the National Cholesterol Education Program's Adult Treatment Panel (NCEP-ATP) III report [18]. MetS was defined as patients with at least two or more of the following four conditions: (1) obesity, BMI ≥25.0 kg/m^2^according to the guidelines of the Japanese Society for the Study of Obesity (waist circumference was not available in this study) [19, 20]; (2) raised BP with a systolic BP (SBP) ≥130 mmHg and/or diastolic BP (DBP) ≥85 mmHg, and/or current treatment for hypertension; (3) hypertriglyceridemia with a TG level ≥150 mg/dL; and (4) low HDL cholesterolemia with a HDL-C level <40 mg/dL in men and <50 mg/dL in women and/or current treatment for dyslipidemia.

### 2.5. Statistical Analysis

All values are expressed as the mean ± standard deviation (SD), unless otherwise specified, and in the cases of parameters with nonnormal distributions (smoking status, TG and FPG), the data are shown as median (interquartile range) values. In all analyses, parameters with nonnormal distributions were used after log transformation. Statistical analysis was performed using IBM SPSS Statistics Version 21 (Statistical Package for Social Science Japan, Inc., Tokyo, Japan). The difference among the non-CVD patients and CVD patients was compared by the *χ* ^2^ test or Student's *t*-test. Multiple linear regression analysis was used to evaluate the contribution of each confounding factor including carotid IMT as well as gender, age, BMI, smoking status, SBP, DBP, presence of antihypertensive medication, non-HDL-C, TG, HDL-C, presence of lipid-lowering medication, FPG, and eGFR for CVD. In addition, areas under the receiver operating characteristic (ROC) curves were determined for each variable to identify the presence of CVD. Areas under the ROC curves are provided with standard errors. An ROC curve is a plot of the sensitivity (true positive) versus 1—specifictiy (false positive) for each potential marker tested. The area under the ROC curve is a summary of the overall diagnostic accuracy of the test. The best markers have ROC curves that are shifted to the left with areas under the curve near unity. Nondiagnostic markers are represented by diagonals with areas under the ROC curves close to 0.5. Likelihood ratios were calculated as the ratios of sensitivity/ (1—specifictiy) (positive likelihood ratio) and (1—sensitivity)/ specificity (negative likelihood ratio). Multiple logistic regression analysis was used to evaluate the contribution of each characteristic for CVD. A value of *P* < 0.05 was considered significant.

## 3. Results

Table 1 shows the clinical characteristics of the 199 non-CVD and 72 CVD patients. Prevalence of male gender and age, prevalence of antihypertensive and lipid-lowering medication, prevalence of low HDL cholesterolemia, and carotid IMT were significantly higher in the CVD patients than those in the non-CVD patients, but HDL-C and eGFR were significantly lower. There were no intergroup differences in BMI, smoking status, SBP, DBP, non-HDL-C, TG, and FPG.

Table 2 shows the relationship between various characteristics and CVD status. Pearson's correlation coefficient showed that carotid IMT as well as male gender, age, prevalence of antihypertensive medication, HDL-C, lipid-lowering medication, and eGFR was significantly related to CVD. Multiple linear regression analysis using the presence of CVD as an objective variable showed that carotid IMT as well as male gender, prevalence of antihypertensive medication, HDL-C, and prevalence of lipid-lowering medication was a significant independent contributing factor.

**Figure 1:**
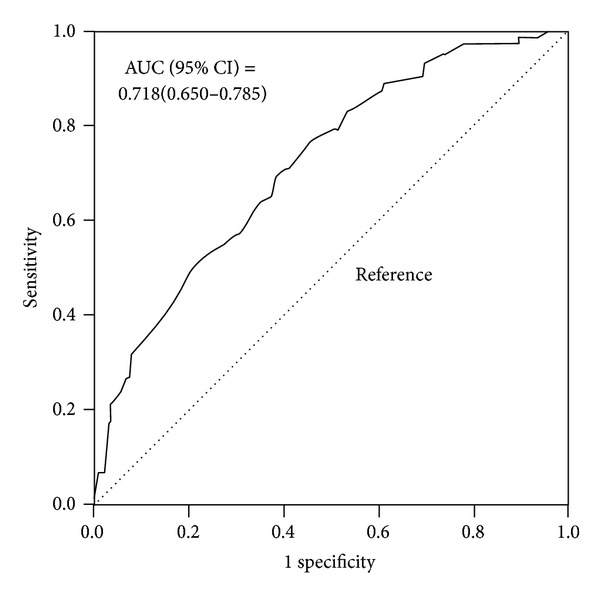
Receiver operating characteristics (ROC) curve. Sensitivity represents the true-positive results and 1—specificity, the false-positive results. The best markers have ROC curves that are shifted to the left with areas under the curve near unity. Nondiagnostic markers are represented by diagonals with areas under the ROC curves close to 0.5. The ROC curve analysis shows that the best marker of CVD was carotid IMT, with an area under the ROC curve of 0.718 (95% confidence interval (CI), 0.650–0.785).

The ROC curve analyses showed that the best marker of CVD was carotid IMT, with an area under the ROC curve of 0.718 (0.650–0.785) (Figure 1). The greatest sensitivity and specificity were obtained when the cut-off value of maximum IMT was set at 0.95 mm (sensitivity = 0.71, specificity = 0.60, and accuracy = 0.627). The positive likelihood ratio value indicates that the odds of CVD would increase 1.76-fold if carotid IMT was ≥0.95 mm, and the negative likelihood ratios indicate the extent to which the odds of CVD would decrease 0.493-fold if carotid IMT was <0.95 mm. 

Table 3 shows the odds ratios (ORs) 95% confidence interval (CI) of CVD with various characteristics in diabetic patients. The unadjusted odds ratio (95% CI) for Low HDL cholesterolemia, carotid IMT ≥0.95 mm, and MetS were 2.36 (1.36–4.08), 3.61 (2.02–6.46), and 1.86 (1.07–3.24), respectively. When adjusted for gender, age, smoking status, non-HDL cholesterol, and eGFR, in model 1 the multivariate-adjusted OR (95% CI) for low HDL cholesterolemia and carotid IMT ≥0.95 mm was 3.24 (1.66–6.30) and 2.30 (1.20–4.43), respectively, and in model 2 the multivariate-adjusted OR (95% CI) for MetS and carotid IMT ≥0.95 mm was 2.45 (1.30–4.62) and 2.41 (1.27–4.60), respectively.

## 4. Discussion

In the present study, we showed that carotid IMT as well as confounding factors was significantly associated with the presence of CVD in Japanese diabetic patients. The ROC curve analysis showed that the area under the curve of carotid IMT was greater than those of the other parameters. The optimal cut-off point to identifying CVD yielded the following values: carotid IMT of ≥0.95 mm. When adjusted for confounding factors, the multivariate-adjusted odds ratio (95% CI) for low HDL cholesterolemia and carotid IMT ≥0.95 mm were 3.24 (1.66–6.30) and 2.30 (1.20–4.43), respectively, and the OR (95% CI) for MetS and carotid IMT ≥0.95 mm was 2.45 (1.30–4.62) and 2.41 (1.27–4.60), respectively. The carotid IMT, an inexpensive and routinely measured clinical variable, might be used as an integrated measure to evaluate the presence of CVD in diabetic patients.

Evidence that measurement of the carotid IMT improves the prediction of the absolute risk of incidental CVD is inconsistent. Many previous studies demonstrated that CVD risk factors (e.g., gender, age, smoking, increased LDL-C level, low HDL-C level, hypertension, and diabetes) are significantly associated with carotid IMT [5, 6, 21, 22]. In prospective studies, the progression of carotid IMT is influenced by CVD risk factors and directly predicts clinical CVD events, independently of classic risk factors [23]. Furthermore, the addition of carotid IMT to the conventional risk factors modestly increased the ability to predict CVD [9, 24]. Also in 900 Japanese outpatients with CVD factors or established atherosclerosis, carotid IMT was a significantly independent predictor after adjustment for risk factors and history of CVD [8]. In a meta-analysis of 37,197 participants followed up for a mean 5.5 years, Lorenz et al. demonstrated that a 0.1 mm absolute difference in carotid IMT was associated with a relative risk of myocardial infarction of 1.15 (95% CI: 1.12–1.17) and a relative risk of stroke of 1.18 (95% CI: 1.16–1.21) [7]. These studies suggest that carotid IMT measurement can assist in identifying people with diabetes who are at a higher risk of developing microvascular and macrovascular complications [23]. In our study, carotid IMT measurements of the risk of CVD events showed that the risk increases with an IMT ≥0.95 mm, and multivariate-adjusted OR (95% CI) for the presence of CVD was 2.41 (1.27–4.60) in patients with diabetes.

Although, recently, conflicting results have been reported on the addictive value of carotid IMT measurements in CVD risk prediction, Lorenz et al. [13] showed that, during a mean followup of 7.0 years among 36,984 participants from 16 studies, the overall hazard ratio of the CVD endpoint was 0.98 (0.95–1.01) when adjusted for vascular risk factors. The association between carotid IMT progression assessed from two ultrasound scans and CVD risk in the general population remains unproven, and no conclusion can be derived for the use of carotid IMT progression as a surrogate marker in clinical trials. Moreover, baseline characteristics and carotid IMT did not significantly influence the association between carotid IMT changes and clinical outcomes [12]. In diabetic individuals, there is no improvement in risk prediction when measurement of carotid IMT is added to the Framingham risk score [25]. These differences may be attributed to differences across studies on carotid IMT measurement (e.g., carotid segments (common, bifurcation, internal), including or excluding carotid plaques), participants' characteristics, cutoff values for risk categories, number of events (small numbers), and end-point definition [26]. We based our analysis on measurements of the mean common carotid IMT because they were available in all studies, they are generally feasible to use in routine clinical practice, and their use has been recommended [17]. In our cross-sectional study, carotid IMT measurement could assist in identifying diabetic people with CVD.

The common carotid artery IMT and inner carotid artery (ICA) IMT were statistically significant predictors of incident CVD, with the ICA IMT having a larger area under the ROC curve (0.756 versus 0.695) [27]. Iglesias Del Sol et al. found that the mean area under the ROC curves of carotid IMT, as a predictor of coronary artery disease, was 0.67 (95% CI: 0.61–0.73) [28]. Irie et al. [29] suggest that the addition of maximum IMT to conventional risk factors significantly improved the prediction ability for severe coronary artery disease in asymptomatic type 2 diabetic patients without history of coronary artery disease (from the area under the curve, 0.67 to 0.79; *P* = 0.039). Also in our study, the mean area under the ROC curves of mean carotid IMT was 0.718 (0.650–0.785) for the presence of CVD among diabetic patients.

We need to be aware of the limitations in interpreting the present results. First, based on its cross-sectional study design, the present result is inherently limited in its ability to eliminate causal relationships between carotid IMT and CVD. Second, since all participants were patients, we could not eliminate the possible effects of underlying diseases (e.g., HbA1c, urine albumin to creatinine ratio) and medication (e.g., salicylic acid, insulin, or oral agent), especially antihypertensive (e.g., angiotensin converting-enzyme inhibitor or angiotensin II receptor blocker agents), and lipid-lowering medications on the results. The use of antihypertensive medications varied from 44.2% in those without CVD to 76.4% in those with CVD, and the use of lipid-lowering medications varied from 6.0% in those without CVD to 27.8% in those with CVD. In this population, the administration rate of antihypertensive medications and lipid-lowering medication is 23.7% and 4.9%, respectively [30]. Third, secondary preventive interventions after obesity, raised BP, dyslipidemia, and diabetes may be successful in reducing the risk factors, thus attenuating the observed association of risk factors with diseases. These points need to be addressed again in prospective population-based studies. 

In conclusions, we report that carotid IMT may be useful for screening diabetic patients with CVD, and its identification may thus be important for risk assessment and treatment of patients. 

## Figures and Tables

**Table 1:** Characteristics of cardiovascular disease and control groups.

Characteristics *N* = 271	Non-CVD *N* = 199	CVD *N* = 72	*P* value*
Male gender, *N* (%)	102 (51.3)	49 (68.1)	0.018
Age (years)	67 ± 10	72 ± 8	<0.001
Body mass index^†^ (kg/m^2^)	23.3 ± 4.0	23.9 ± 4.3	0.313
Obesity, *N* (%)	54 (27.1)	24 (33.3)	0.363
Smoking status (pack·year)	0 (0–520)	300 (0–845)	0.092
Systolic blood pressure (mmHg)	140 ± 23	137 ± 20	0.389
Diastolic blood pressure (mmHg)	79 ± 14	77 ± 13	0.198
Antihypertensive medication (%)	88 (44.2)	55 (76.4)	<0.001
Raised blood pressure, *N* (%)	159 (79.9)	61 (84.7)	0.482
Non-HDL cholesterol (mg/dL)	141 ± 43	136 ± 45	0.460
Triglycerides (mg/dL)	90 (67–146)	96 (65–129)	0.590
Hypertriglyceridemia, *N* (%)	48 (24.1)	13 (18.1)	0.327
HDL cholesterol (mg/dL)	55 ± 19	49 ± 15	0.015
Lipid-lowering medication, *N* (%)	12 (6.0)	20 (27.8)	<0.001
Low HDL cholesterolemia, *N* (%)	69 (34.7)	40 (55.6)	0.003
Fasting plasma glucose (mg/dL)	166 (118–229)	137 (105–186)	0.082
eGFR^‡^ (mL/min/1.73 m^2^)	74.9 ± 21.0	66.6 ± 22.3	0.005
Carotid IMT (mm)	0.92 ± 0.20	1.11 ± 0.28	<0.001

CVD: cardiovascular disease; HDL: high-density lipoprotein; eGFR: estimated glomerular filtration ratio; IMT: intima-media thickness. ^†^Body mass index was calculated using weight in kilograms divided by the square of the height in meters. ^‡^eGFR = 194 × Cr^−1.094^ × Age^−0.287^ × 0.739 (if female). Data presented are mean ± standard deviation. Data for smoking status, triglycerides, and fasting plasma glucose were skewed, are presented as median (interquartile range) values, and were log transformed for analysis.

**Table 2:** Relationship between various confounding factors and CVD.

Characteristics *N* = 271	Pearson's correlation coefficient *r* (*P* value)	Multiple linear regression analysis *β* (*P* value)
Male gender, %	0.149 (0.014)	0.197 (0.006)
Age (years)	0.238 (<0.001)	0.090 (0.165)
Body mass index (kg/m^2^)	0.062 (0.313)	0.033 (0.587)
Smoking status (pack·year)	0.102 (0.092)	−0.009 (0.896)
Systolic blood pressure (mmHg)	−0.052 (0.389)	−0.103 (0.136)
Diastolic blood pressure (mmHg)	−0.079 (0.198)	0.016 (0.822)
Antihypertensive medication (%)	0.285 (<0.001)	0.194 (0.001)
Non-HDL cholesterol (mg/dL)	−0.045 (0.460)	0.024 (0.725)
Triglycerides (mg/dL)	−0.033 (0.590)	−0.132 (0.059)
HDL cholesterol (mg/dL)	−0.148 (0.015)	−0.191 (0.001)
Lipid-lowering medication (%)	0.298 (<0.001)	0.284 (<0.001)
Fasting plasma glucose (mg/dL)	−0.106 (0.082)	−0.057 (0.296)
eGFR (mL/min/1.73 m^2^)	−0.170 (0.005)	−0.058 (0.312)
Carotid IMT (mm)	0.353 (<0.001)	0.259 (<0.001)

Data for smoking status, triglycerides, and fasting plasma glucose were skewed and log transformed for analysis.

**Table 3:** Determinants of CVD versus non-CVD from simple and multiple logistic regression models.

Characteristics *N* = 271	Unadjusted odds ratio (95% CI)	Adjusted odds ratio (95% CI)
Model 1		
Obesity		
Odds ratio (95% CI)	1.34 (0.75–2.40)	1.58 (0.77–3.22)
Raised blood pressure		
Odds ratio (95% CI)	1.40 (0.67–2.89)	1.32 (0.57–3.08)
Hypertriglyceridemia		
Odds ratio (95% CI)	0.69 (0.35–1.37)	0.52 (0.22–1.24)
Low HDL cholesterolemia		
Odds ratio (95% CI)	**2.36** (**1.36**–**4.08**)	**3.24** (**1.66**–**6.30**)
Carotid IMT ≥ 0.95 mm		
Odds ratio (95% CI)	**3.61 **(**2.02**–**6.46**)	**2.30 **(**1.20**–**4.43**)
Model 2		
Metabolic syndrome		
Odds ratio (95% CI)	**1.86 **(**1.07**–**3.24**)	**2.45 **(**1.30**–**4.62**)
Carotid IMT ≥ 0.95 mm		
Odds ratio (95% CI)	**3.61 **(**2.02**–**6.46**)	**2.41 **(**1.27**–**4.60**)

^†^Adjusted for gender, age, smoking status, non-HDL cholesterol, and eGFR. Data for smoking status was skewed and log transformed for analysis.
